# Systemic Inflammation and Cardiovascular Comorbidity in Psoriasis Patients: Causes and Consequences

**DOI:** 10.3389/fimmu.2018.00579

**Published:** 2018-04-05

**Authors:** Wolf-Henning Boehncke

**Affiliations:** ^1^Department of Pathology and Immunology, Faculty of Medicine, University of Geneva, Geneva, Switzerland; ^2^Division of Dermatology and Venereology, Geneva University Hospitals, Geneva, Switzerland

**Keywords:** psoriasis, coronary heart disease, stroke, atherosclerosis, insulin resistance, endothelial dysfunction, mortality

## Abstract

Psoriasis is a common inflammatory skin disease characterized by the appearance of red scaly plaques that can affect any part of the body. High prevalence, chronicity, disfiguration, disability, and associated comorbidity make it a challenge for clinicians of multiple specialties. Likewise, its complex pathogenesis, comprising inflammation, hyperproliferation, and angioneogenesis, intrigues numerous scientific disciplines, namely, immunology. From a clinical perspective, the severity of psoriasis is highlighted by its increased mortality, with cardiovascular diseases contributing the highest excess risk. From a scientific point of view, psoriasis has to be considered a systemic inflammatory condition, as blood biomarkers of inflammation are elevated and imaging techniques document sites of inflammation beyond the skin. While the association of psoriasis with cardiovascular diseases is now widely accepted, causes and consequences of this association are controversially discussed. This review comments on epidemiologic, genetic, and mechanistic studies that analyzed the relation between psoriasis and cardiovascular comorbidity. The hypothesis of psoriasis potentially being an independent cardiovascular risk factor, driving atherosclerosis *via* inflammation-induced endothelial dysfunction, will be discussed. Finally, consequences for the management of psoriasis with the objective to reduce the patients’ excess cardiovascular risk will be pointed out.

## Introduction

Psoriasis is among the few non-communicable diseases the World Health Organization identified as a major global health problem (1). It is a common inflammatory skin disease, affecting around 2% of the population in Western countries (2). Its clinical hallmark are red scaly plaques that can affect any part of the body, but preferentially appear over elbows and knees as well as on the scalp, the umbilical, and perianal region. These lesions reflect the principal pathogenetic mechanisms underlying psoriasis, namely, inflammation, hyperproliferation, and angioneogenesis. To date, psoriasis is considered an immune-mediated disease, exhibiting an intense cross talk between components of the innate and the adaptive immune system, which then trigger the epidermal changes (3). Psoriatic inflammation goes more than skin deep, as signs of inflammation can readily be detected at sites outside the skin (4).

Comorbidity is a common feature of many immune-mediated inflammatory diseases across different medical specialties, including model diseases such as rheumatoid arthritis (5), inflammatory bowel disease (6), or multiple sclerosis (7). A wealth of data documents the association of psoriasis with numerous comorbid diseases, including cardiometabolic, gastrointestinal, and kidney diseases as well as malignancies, infections, and mood disorders (8). To this end, it is still controversial whether psoriatic arthritis should be considered an extracutaneous manifestation of psoriasis or as a separate entity, and thus comorbidity (9). Among the comorbid conditions, cardiovascular diseases are of particular importance, as they often directly impact the patients’ mortality (10).

In the case of rheumatoid arthritis, currently available evidence is strong enough to allow the issue of recommendations that substantially change prevention and treatment goals for cardiovascular risk factors in these patients (11). And while the association with cardiovascular diseases also among psoriasis patients is now widely accepted, causes and consequences of this association are controversially discussed (12–14). This review comments on epidemiologic, genetic, and mechanistic studies that analyzed the relation between psoriasis and cardiovascular comorbidity. The hypothesis of psoriasis potentially being an independent cardiovascular risk factor, driving atherosclerosis *via* inflammation-induced endothelial dysfunction, will be discussed. Finally, consequences for the management of psoriasis with the objective to reduce the patients’ excess cardiovascular risk will be pointed out.

## Epidemiology

One of the first systematic analyses on disease concomitance based on more than 40,000 cases identified an association of psoriasis not only with cardiovascular disease but also with diseases that represent risk factors for atherosclerosis, such as diabetes mellitus or obesity. The authors concluded that a distinct pattern of associated diseases exists in patients with psoriasis and suggest a genetically determined selection (15). Since then, multiple epidemiologic studies have addressed the issue of cardiovascular comorbidity in psoriasis patients. And while some groups concluded that such a link does not exist (16–18), many others were able to reproduce this association recently summarized elsewhere (8, 19) (Table 1). Based on the currently available evidence, most experts agree that the association of psoriasis with cardiovascular comorbidity is real (20). This is also reflected by and justifies the inclusion of advice on the management of psoriasis patients with such comorbidities in guidelines and treatment recommendations (21, 22).

**Table 1:** Summary of studies analyzing the association between psoriasis and major adverse cardiovascular events.

Reference	Study characteristics	Key finding
Dowlatshahi et al. (16)	Population-based study (262 patients with mostly mild psoriasis, and 8,009 controls)	No increased risk for cardiovascular events

Parisi et al. (17)	Cohort study (48,000 patients and 200,000 controls)	No association of psoriasis with cardiovascular disease

Egeberg et al. (18)	Nationwide cohort study (adult population of Denmark)	Slight increase of myocardial infarction in patients with severe psoriasis

Armstrong et al. (23)	Systematic review and meta-analysis (220,000 patients and 10 mio controls)	Increased risk for myocardial infarction, stroke, and cardiovascular mortality among psoriasis patients

Gaeta et al. (24)	Meta-analysis (1.8 mio patients and 43 mio controls)	Increased risk for myocardial infarction and cardiovascular mortality among psoriasis patients

Gu et al. (25)	Meta-analysis of cohort studies (6.2 mio individuals overall)	Increased risk for myocardial infarction, stroke, and cardiovascular mortality among psoriasis patients

Horreau et al. (26)	Systematic literature review (324,000 patients and 5.3 mio controls)	Increased risk for myocardial infarction and stroke among psoriasis patients

Miller et al. (27)	Meta-analysis (500,000 patients and 29 mio controls)	Increased risk for cardiovascular disease among psoriasis patients

Pietrzak et al. (28)	Review (360,000 patients and 9.2 mio controls)	Increased risk for cardiovascular events among psoriasis patients

Samarasekera et al. (29)	Systematic review and meta-analysis (480,000 patients and 10 mio controls)	Increased risk for myocardial infarction, stroke, and cardiovascular mortality among psoriasis patients

Xu et al. (30)	Meta-analysis of cohort studies (326,000 patients and 5.2 mio controls)	Increased risk for myocardial infarction and stroke among psoriasis patients

The fact that cardiovascular diseases are associated with psoriasis leads to the question on why that might be so. A strong argument in favor of an indirect link comes from the observation that psoriasis is associated with numerous conditions representing major cardiovascular risk factors in their own right (Table 2). In fact, the very first publication on psoriasis comorbidity reported an association with diabetes mellitus as early as 1897 (31). Also, the study by Henseler and Christophers cited above documents diabetes mellitus and obesity as concomitant conditions (15). Finally, Takeshita et al. recently summarized the evidence for obesity, hypertension, diabetes mellitus, dyslipidemia, and the metabolic syndrome as comorbid diseases in psoriasis (8), with the metabolic syndrome essentially representing the combined appearance of obesity, hypertension, insulin resistance, and dyslipidemia (32). These conditions are not only well-established major cardiovascular risk factors and therefore incorporated in the Framingham’s Cardiovascular Disease Risk Scores used to predict cardiovascular risk in the general population (33), some of them, namely, smoking (34) and obesity (35), are also risk factors for the development of psoriasis.

**Table 2:** Synopsis of arguments in favor or against the hypothesis of psoriasis representing an independent cardiovascular risk factor.

Domain	Psoriasis may be an independent cardiovascular risk factor	Psoriasis may *not* be an independent cardiovascular risk factor
Epidemiology	Dose effect: Population-based studies document a higher cardiovascular risk among patients with severe compared to those with mild psoriasis. The risk is also higher in patients with longer disease duration. In case–control studies, surrogate markers for increased cardiovascular risk are associated with psoriasis after thorough control for confounding factors.	Conventional cardiovascular risk factors such as several or even all components of the metabolic syndrome are associated with psoriasis throughout all age groups

Genetics	A comprehensive assessment of the catalog of genome-wide association studies shows that the genetic control of psoriasis is almost completely independent from both the metabolic syndrome and coronary heart disease	There may be some shared susceptibility loci between psoriasis and its comorbidities
		A missense mutation in the insulin-responsive peptidase links psoriasis to hypertension and diabetes mellitus

Pathophysiology	Remarkable similarities exist between the inflammatory processes in psoriatic and atherosclerotic plaques: • insulin resistance • endothelial dysfunction • T-lymphocyte driven • neutrophils involved • monocytes/macrophages involved • platelets involved	The exact role of several potentially shared components has yet to be established: • macrophages • IL-17A

Evidence in favor of psoriasis as an independent cardiovascular risk factor comes from studies showing a “dose effect” of psoriasis on the patients’ cardiovascular risk (Table 2). A landmark study in this regard was conducted by Gelfand et al. who used the General Practice Research Database from 1987 to 2002, comprising prospective data collected from general practitioners in Great Britain. After adjusting for major cardiovascular risk factors, such as hypertension, diabetes mellitus, and hyperlipidemia, they found a slightly elevated adjusted relative risk for myocardial infarction among patients with mild psoriasis, and a substantially elevated adjusted relative risk among patients with severe psoriasis (36). Two meta-analyses also came to the conclusion that the cardiovascular risk of psoriasis patients correlates with the severity of their disease (29, 23). In line with this hypothesis, longer duration of psoriasis has also been associated with increased cardiovascular risk for the patients (37, 38). Another landmark study was performed by Ludwig et al. who quantified coronary artery calcification *via* CT scans, using the well-established Agatston score, among hospitalized psoriasis patients, thus suffering from severe psoriasis (39). Their scores were compared to controls matched for all major cardiovascular risk factors. The study showed highly significantly elevated coronary artery calcification, so that the study had to be stopped before the originally calculated number of patients had been included. This observation is even more alarming, as only patients with a negative history for current or previous heart problems were included.

The clinical relevance of psoriasis as an independent cardiovascular risk factor was quantified by Mehta et al. in a cohort study of severe psoriasis patients. There, the attributable risk of severe psoriasis on major cardiovascular events, i.e., namely, myocardial infarction and stroke, over a 10-year period was found to be around 6% (40). Others found the increased risk of such events associated with psoriasis to be comparable with that conferred by diabetes mellitus alone (41) or by rheumatoid arthritis (42). This is remarkable as the former is a well-accepted major cardiovascular risk factor, and the association of the latter with an increased cardiovascular risk already led to specific recommendations on how to address this extra risk, as pointed out above.

Taken together, despite few studies that did not show statistically significant associations between psoriasis and major cardiovascular events, a majority of studies using different methodical approaches suggests not only an association of psoriasis with cardiovascular diseases but also provides evidence for psoriasis as an independent cardiovascular risk factor (Table 2).

Noteworthy, chronic skin inflammation as such is not sufficient to explain the role of psoriasis as an independent cardiovascular risk factor, as a recent systematic review and meta-analysis failed to provide evidence for atopic dermatitis, another common chronic inflammatory skin disease, exhibiting comparable effects (43). A major epidemiologic study documented even the inverse phenomenon, namely, a rather significantly decreased risk for myocardial infarction, stroke, or cardiovascular death (44). Therefore, genetic and pathogenetic analyses are needed to understand this association.

## Genetics

One possibility to explain the association of psoriasis with its comorbid conditions in general and cardiovascular disease in particular is that these entities share common genetics. A genetic predisposition of psoriasis can already be postulated based on the facts that many patients have a positive family history and that the concordance rate among monozygotic twins is much higher compared to dizygotic twins (45). The first psoriasis susceptibility locus (PSORS-1) identified is located on the short arm of chromosome 6 in the region coding for major histocompatibility complex (MHC) molecules. It is still the most reproducible among all PSORS loci identified and may explain up to 50% of the heritability of psoriasis (46). Namely genome-wide association studies (GWASs) shed more light on the genetics of psoriasis and allowed to identify at least 50 regions on the human genome which harbor at least 1 and sometimes more than 1 potential candidate gene associated with psoriasis. This database allows to group the respective genes according to pathways, with antigen presentation, interleukin-23 (IL-23) signaling, T-lymphocyte development and polarization, innate immunity, and negative regulators of immune responses as the key axes (47).

Following up on the notion that chronic skin inflammation as such does not suffice to explain increased cardiovascular risk, a GWAS showed some overlap between susceptibility loci for psoriasis and atopic dermatitis. This association was mediated by a combination of shared and opposing alleles, with the most significant effects operating in opposing directions, thus arguing in favor of distinct pathogenetic pathways of these diseases (48). This notion is supported by a much smaller study from Japan that observed only marginal associations between atopic dermatitis and susceptibility single-nucleotide polymorphisms for psoriasis (49). By contrast, rheumatoid arthritis as another chronic inflammatory disease with known cardiovascular comorbidity does exhibit a genetic architecture not too different from psoriasis: genetic variants in the MHC region account for more than 60% of the known genetic heritability of rheumatoid arthritis. Other associated genes are involved in the function of T-lymphocytes and monocytes representing the adaptive and the innate immune system, respectively. 35 rheumatoid arthritis risk loci gene products can be mapped to signaling pathways in T-lymphocytes and antigen-presenting cells (50). These include components of the tumor necrosis factor alpha (TNF-α) signaling cascade, the potential relevance of this finding will be discussed below.

Genetic variation and pathways in atherosclerosis do not show obvious overlap with the genetic signature of psoriasis or rheumatoid arthritis. Kessler et al. recently assigned 30 of 65 loci associated with coronary artery disease to 6 pathways with known pathophysiological roles in this disease, the function of the other 35 candidate genes is not sufficiently known to allow allocation to a particular pathway. These six pathways comprise inflammation, triglycerides, LDL cholesterol, blood pressure, vascular remodeling, and nitric oxide signaling (51).

Several groups have further analyzed the hypothesis of psoriasis and its comorbidities being genetically linked, following up on the observation that GWASs have shown overlap in the genetic susceptibility to different pathologies, namely, immune-mediated diseases (52). Ellinghaus et al. identified seven shared susceptibility loci for psoriasis and Crohn’s disease; they went on to demonstrate a genetic overlap between five seronegative inflammatory diseases, namely psoriasis, Crohn’s disease, ankylosing spondylitis, primary sclerosing cholangitis, and ulcerative colitis (53, 54). An observation pointing toward a genetic link between psoriasis and cardiovascular comorbidity was made by Cheng et al. who interpreted a missense mutation in the insulin-responsive aminopeptidase LNPEP as a potential link, namely between psoriasis, hypertension, and diabetes mellitus (55) (Table 2). The importance of insulin resistance in this context will be discussed in detail below. On the other hand, a comprehensive assessment of the catalog of GWASs by Gupta et al. shows that the genetic control of psoriasis is almost completely independent from both the metabolic syndrome and coronary heart disease (Table 2). To prove reliability of this approach, the authors were able to identified 10 common loci for the metabolic syndrome and coronary heart disease, using exactly the same data set (56).

Taken together, although some genetic overlap between psoriasis and several of its comorbid conditions exists, the observed association of psoriasis with cardiovascular comorbidity cannot satisfyingly be explained by shared genetics (Table 2).

## Pathogenesis

### Common Pathways of Psoriatic and Atherosclerotic Plaque Formation

To this end, epidemiological evidence in favor of psoriasis being associated with cardiovascular comorbidity and potentially functioning as an independent cardiovascular risk factor has been summarized. As genetic overlap cannot satisfyingly explain the excess cardiovascular risk of patients with severe psoriasis, mechanistic studies are needed to further clarify the link.

Often, animal models are a good starting point to unravel pathogenetic mechanisms. With regard to psoriasis, older models often highlighted the role of the adaptive immune system (57), while novel approaches such as mannan-induced psoriasis shed more light on the crucial role of the innate immune system (58). Few studies have been performed to analyze the link between psoriasis and its cardiovascular comorbidity. One such approach takes advantage of the capacity of the TLR7 agonist imiquimod to induce a psoriasis-like inflammation in murine skin. Jin et al. succeeded in inducing a state of systemic inflammation through topical application of imiquimod onto interleukin-10-deficient mice, but noticed a decrease of body weight in these mice, while psoriasis patients show a trend toward obesity (59). Shibata et al. studied imiquimod-induced psoriasiform inflammation in mice deficient of adiponectin, an anti-inflammatory adipokine, and found a more severe phenotype in these knockout mice. Furthermore, intraperitoneal injections of adiponectin had a therapeutic effect in adiponectin knockout mice (60). While this study explored an indirect link between psoriasis and cardiovascular disease *via* metabolic factors, the only direct link the author is aware of comes from a study in the type-II collagen-specific antibody-induced psoriatic arthritis model. Using this model, Sherlock et al. demonstrated that IL-23 drives inflammation in the aortic root through activation of CD3^+^CD4^−^CD8^−^ T-lymphocytes (61).

Atherosclerosis as a key process in cardiovascular diseases has long been recognized as an inflammation-driven phenomenon (62, 63). This is also true for psoriasis (64, 65). The cardiologist Späh was among the first who discussed a potentially common inflammatory pathway and the idea of an integrated treatment approach (66). He stressed altered endothelial function and subsequent recruitment of leukocytes, primarily T-lymphocytes, to developing lesions as a shared early step in the process of plaque formation in atherosclerosis and psoriasis. Lymphocyte extravasation has indeed been studied in detail with the intention to develop targeted therapies for psoriasis, but to date, none of the potential candidates was found to be sufficiently effective to validate development into a marketed drug (67, 68). Meanwhile, many more shared mechanisms of atherosclerosis and psoriasis have been studied in detail.

Elaborating on the observations described above, a role namely for T-helper-1 lymphocytes (TH1 lymphocytes) has been established in atherosclerosis as well as psoriasis (Table 2). Although TH1 as well as TH2 lymphocyte responses can contribute to atherosclerosis, several lines of evidence suggest a predominant role of TH1 lymphocytes. These include the TH1 phenotype and function of most T-cell clones derived from atherosclerotic plaques as well as immunohistochemical studies on such plaques (69–71). Subsequently, high circulating levels of TH17 lymphocytes and IL-17 in patients with acute coronary syndrome, a positive correlation of IL-17 with levels of high-sensitivity C-reactive protein and IL-6 (predicting an increased risk of myocardial infarction) in those patients, and the observation that IL-17 inhibition in mice significantly reduces the size of atherosclerotic plaques, were all interpreted as indicators for a role of TH17 lymphocytes in atherosclerosis (72–74). Similarly, psoriasis was initially thought to be a prototypical TH1 lymphocyte-mediated disease, with these cells activating macrophages, neutrophils and CD8^+^ cytotoxic lymphocytes (75). Then, the role of TH17 lymphocytes was stressed in the light of clinical studies documenting the high clinical efficacy of therapies targeting the IL-17 pathway (76).

While the role of the adaptive immune system in the pathogenesis of psoriasis has been thoroughly investigated ever since the accidental observation of the therapeutic efficacy of cyclosporine A in 1979 (77), interest in the important role of the innate immune system has only recently experienced a renaissance (Table 2). Evidence for the contribution of neutrophils, which are predominant in pustular forms, but readily detectable also in chronic plaque type psoriasis, where they form the so-called Munro’s abscesses within the epidermis, comes from *in vivo* studies as well as organotypic 3D models and has recently been reviewed elsewhere (78). Further supporting the role of neutrophils is the clinical observation by Reich et al., who reported that psoriasis treatment with the anti-IL17A antibody secukinumab resulted in the near total elimination of intraepidermal IL-17-positive neutrophils as an early therapeutic effect (79). Neutrophils are equally important in atherosclerosis, as they interact with damaged endothelium, augment leukocyte recruitment *via* secretion of chemotactic mediators, and promote the development of foam cells, a macrophage subset driving atherosclerosis (80). Neutrophil localization to developing atherosclerotic plaques has been demonstrated in mouse models (81, 82) and human atherosclerotic lesions (83); their presence in occlusive thrombi and culprit lesions of acute coronary syndrome patients suggests a role in atherosclerotic progression (84).

As for neutrophils, involvement of monocytes and macrophages has readily been demonstrated in both diseases (Table 2). These are regularly detectable in psoriatic lesions (85). Using a mouse model where the psoriatic phenotype is induced by topical application of the immunomodulator imiquimod, Costa et al. demonstrated that induction of the phenotype depends exclusively on hematopoietic cells. Using conditional knockout mouse strains, the active contribution of monocytes and macrophages on disease propagation and exacerbation was shown (86). With regard to atherosclerosis, Tabas and Lichtman recently reviewed how macrophages can be programmed for functions on a spectrum from inflammatory and host defense to resolution and repair in atherosclerotic plaques (87). In general, inflammatory macrophages carry out processes that promote atherosclerosis progression, including plaque necrosis and thinning of a protective fibrous cap. By contrast, resolving macrophages carry out functions that can suppress plaque progression and promote plaque regression, including clearing dead cells and secreting collagen that can form a protective scar over the lesion (88).

Besides these vigorously studied cells of the adaptive immune system, platelets seem to also actively contribute to atherosclerosis and psoriasis (Table 2). Platelets are widely known to have prominent functions in hemostasis and thrombosis, but their involvement in immune and inflammatory processes is now more and more recognized (89). Their potential to influence such process is not surprising, given their capacity to release a plethora of mediators and to interact with numerous cells and tissues through a variety of adhesion molecules. In psoriasis, platelet activation can be used to monitor disease activity through quantification of platelet activation markers in the patients’ blood (90). Mechanistically, it is thought that activated platelets facilitate leukocyte extravasation (91). Similar effects link platelets to atherosclerosis (92, 93). The clinically relevant role of platelets in this context is underlined by the success of platelet inhibitory drugs in treating and preventing acute arterial thromboembolic events (94).

In recent years, a factor attracting particular attention in the context of psoriatic as well as atherosclerotic inflammation is IL-17A. Its role as a major driver of psoriatic inflammation is now well accepted and underlined by the numerous lines of evidence, including the development of a psoriatic phenotype in mice overexpressing IL-17A in the epidermis (95), or the high efficacy of IL-17A-blocking biologics in the treatment of psoriasis (96). By contrast, deciphering the exact role of IL-17A in atherosclerosis remains a challenging task (Table 2). Numerous studies documented pro-atherogenic effects. For example, in a hypercholesterolemic animal model, IL-17A inhibition reduced atheroma area and stenosis. At the molecular level, expression of the chemokine CCL5 (CC chemokine ligand 5), the cytokines IL-6 and TNF-α, several adhesion molecules including vascular cell adhesion molecule 1, and the pro-thrombotic molecule TF was reduced (74). Complementary experiments were conducted in murine models, where addition of exogenous IL-17A-stimulated pathological changes associated with increased plaque instability, while IL-17A inhibition resulted in regression of atherosclerosis (97–99). *Ex vivo* studies on human plaque fragments showed that exposure to IL-17A induced pro-inflammatory, pro-thrombotic, plaque destabilizing, and cell-attracting effects (100). On the other hand, there is also some evidence suggesting that IL-17A may have anti-atherosclerotic effects, as another mouse model characterized by increased IL-17A expression showed significantly smaller atherosclerotic lesions. In the same publication, an association between IL-17A expression and plaque stability in human carotid artery plaques was reported (101). Moreover, a study from Simon et al. on almost 1,000 patients with acute myocardial infarction demonstrated that low serum levels of IL-17A are associated with a higher risk for major cardiovascular events (102). Overall, many experts lean toward the concept of IL-17A being primarily pro-atherogenic, although some uncertainty persists.

### Psoriatic Inflammation as a Driver for Atherosclerosis

Thus, over the last decade, multiple shared pathogenetic mechanisms have been identified in psoriatic and atherosclerotic plaque formation. These similarities do not, however, explain why psoriasis might actually represent an independent cardiovascular risk factor, as suggested by the majority of epidemiologic studies. Of major importance in this regard was the notion that psoriasis cannot be regarded as isolated cutaneous inflammation, but rather represents a chronic systemic inflammatory disease. To this end, several groups have identified biomarkers of inflammation in the blood of psoriasis patients which correlate with psoriasis severity, such as C-reactive protein (103), erythrocyte sedimentation rate (104), and the platelet activation marker P-selectin (90). Documentation of vascular inflammation through (18)F-fluorodeoxyglucose positron emission tomography computed tomography (PET-CT) in psoriasis patients, pioneered by Mehta and co-workers, points into the same direction (4). More recently, the group demonstrated that psoriasis severity associates with aortic vascular inflammation detected by that method (93), suggesting that psoriatic inflammation affects blood vessels and induces inflammation in the vessel walls. Studies in mouse models confirm that chronic skin-specific inflammation can indeed induce vascular inflammation (94).

**Figure 1:**
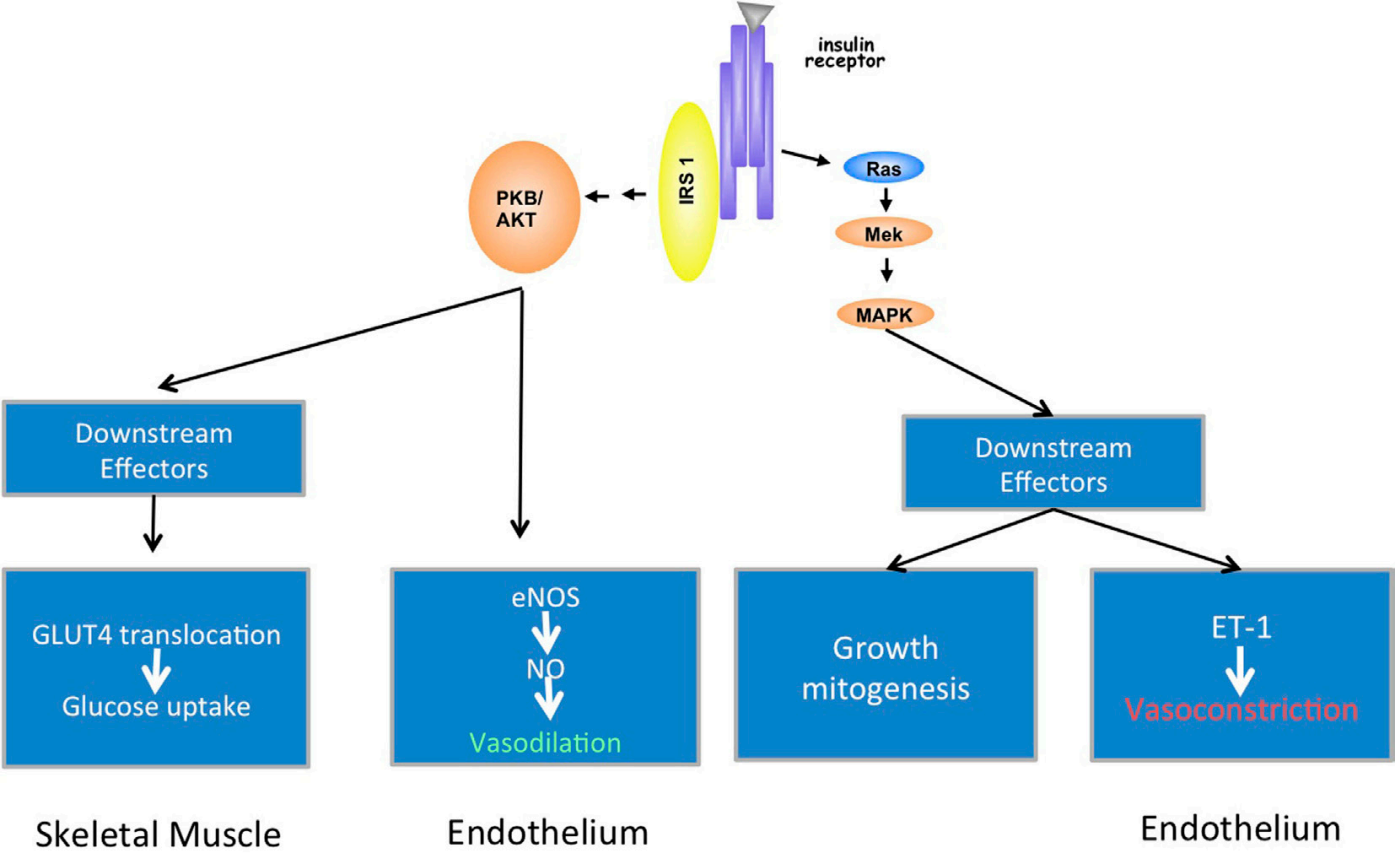
Metabolic and vascular effects of insulin. Under physiologic conditions, the activated insulin receptor on endothelial cells leads to phosphorylation of endothelial nitric oxide synthase (eNOS) *via* activation of insulin receptor substrate (IRS) and phosphoinositide-3-kinase (a). This results in vasodilation *via* NO production. This branch also regulates GLUT4 translocation and glucose uptake in muscle cells. The mitogen-activated protein kinase (MAPK) pathway controls secretion of vasoconstrictive ET-1 in endothelial cells, and cell growth and mitogenesis in cells at large. Inflammation reduces NO production *via* blocking insulin receptor signaling at the level of the IRS-1 (b).

The pathogenetic link between psoriasis and cardiovascular comorbidity is likely provided through insulin resistance and endothelial dysfunction, as these are known drivers for atherosclerosis (105). Insulin resistance is typically defined as decreased sensitivity to metabolic actions of insulin that promote glucose disposal. This is not only an important feature of diabetes mellitus but also a prominent component of cardiovascular disorders, which are characterized by endothelial dysfunction (106). Conversely, endothelial dysfunction is also present in diabetes mellitus (107). In addition to its essential metabolic actions, insulin has important vascular actions that involve stimulation of the production of nitric oxide from endothelium, leading to vasodilation (Figure 1A). This effect has metabolic consequences, too, as increased blood flow ultimately leads to augmented glucose disposal in skeletal muscle (108). On the other hand, insulin signaling in endothelial cells regulates secretion of the vasoconstricting factor endothelin-1. Inflammation shifts this equilibrium as it induces insulin resistance *via* cytokines, which alter insulin signaling in endothelial cells, ultimately reducing the production of vasodilating nitric oxide and inducing endothelial dysfunction (109) (Figure 1B). Noteworthy, TNF-α, a central cytokine in the pathogenesis of many chronic inflammatory diseases including psoriasis, is a major insulin antagonist (110).

**Figure 2:**
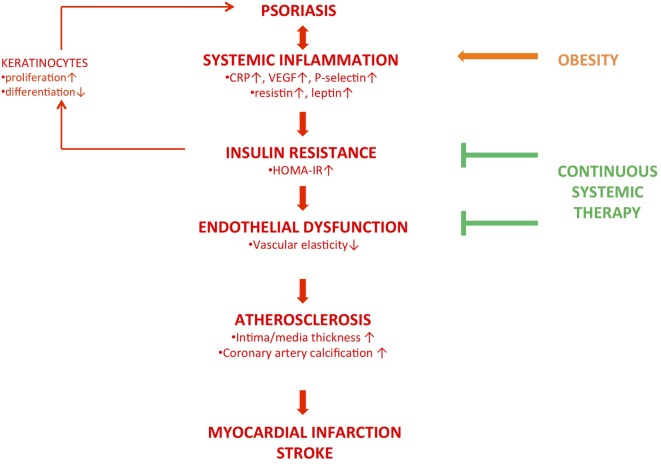
The concept of the “psoriatic march.” This hypothesis suggests that psoriasis is a systemic inflammatory condition, as numerous biomarkers of inflammation are elevated in the patients’ blood compartment. Functional consequences are insulin resistance, evidenced by an increased HOMA-IR (homeostasis assessment of insulin resistance), and endothelial dysfunction, resulting in increased vascular stiffness. This provides the basis for atherosclerosis, observable through analysis of vessel wall composition *via* CTs or ultrasound. Depending on the sites of atherosclerosis, major cardiovascular events such as myocardial infarction and stroke result from this. This “backbone” (red, bold) may be developed further by adding additional “modules”: insulin resistance has been shown to alter epidermal homeostasis (red, fine). Obesity, causing a state of systemic inflammation as well, is a known risk factor for psoriasis and may induce the phenotype (orange, bold). Whether systemic anti-inflammatory therapy is capable of reducing the patients’ cardiovascular risk through reducing insulin resistance and endothelial dysfunction is still a matter of debate (green).

The concept of the “psoriatic march” provides a framework to explain how psoriatic inflammation drives cardiovascular comorbidity *via* atherosclerosis independently from the presence of additional cardiovascular risk factors (111, 112) (Figure 2). According to this concept, psoriasis is a chronic systemic inflammatory disorder, as evidenced by elevated biomarkers of systemic inflammation. Noteworthy, not only classical markers for systemic inflammation have been shown to be elevated but also resistin and leptin (113, 114). These belong to a family of mediators secreted by adipocytes called adipokines. Resistin and leptin are insulin antagonizing adipokines. Collectively, the adipokine milieu in the blood of psoriasis patients is strikingly similar to that of prediabetic individuals and is a hint toward a state of insulin resistance. The diagnosis of insulin resistance is based on the so-called homeostasis model assessment of insulin resistance (HOMA-IR) index, calculated on the basis of a blood test, or an oral glucose tolerance test (115, 116). Using these approaches, two cross-sectional studies demonstrated that psoriasis patients exhibit insulin resistance at clinical levels (113, 117). Besides the insulin-antagonizing effects, numerous adipokines in general and leptin in particular may drive atherosclerosis through immunomodulation, including the upregulation of adhesion molecules on endothelial cells (118). At the level of endothelial cells, insulin resistance is thought to induce endothelial dysfunction *via* the pathway described above, resulting in vascular stiffness at the functional level. Indeed, several groups found evidence for endothelial dysfunction, using ultrasound methods. In particular, flow-mediated vascular dilation was impaired (119–122). In one of these studies, insulin resistance was assessed as well *via* the HOMA-IR and found to be significantly higher when compared to non-psoriatic controls, again stressing the link between insulin resistance and endothelial dysfunction in psoriasis (122). This cascade drives atherosclerosis, which ultimately causes cardiovascular diseases such as myocardial infarction and stroke.

Although not in the focus of this review, it is interesting to note that inflammation-induced insulin resistance may well help to explain the altered epidermal homeostasis observed in the epidermis of psoriatic plaques (123, 124), a phenomenon that might have implications beyond psoriasis (125).

## Consequences for the Management of Psoriasis

### Monitoring

Most observations discussed so far support the notion that cardiovascular comorbidity is a clinically relevant problem for patients suffering from severe psoriasis. In spite of this, several studies document that these patients are not adequately monitored and treated for cardiovascular risk factors. A survey conducted between October 2010 and April 2011 among primary care physicians and cardiologists showed that most of the responding physicians (251 of 1,200 questionnaires sent out were returned) did not routinely screen psoriasis patients for cardiovascular risk factors (126). A cross-sectional study based on National Ambulatory Medical Care Survey data from 2005 to 2009 demonstrated that less than half of the psoriasis patients were screened for at least one cardiovascular risk factor (127). Finally, in a cross-sectional study of patients with hypertension in Great Britain, psoriasis patients were more likely to have uncontrolled hypertension compared with non-psoriatic individuals (128). This is in contrast to recommendations from numerous organizations and societies, who unanimously suggest such a screening (129–131). Takeshita et al. recently summarized what most experts currently agree upon, much of it being identical to what is comprised in current recommendations for the general adult population (132, 133): traditional cardiovascular risk factors should be evaluated. These include total and high-density lipoprotein cholesterol, systolic blood pressure, use of antihypertensive therapy, diabetes, and current smoking. In addition, lifestyle interventions, such as weight loss and smoking cessation, should be encouraged among patients who are obese and who are current smokers, respectively. A controlled clinical trial published a decade ago already showed the potential advantages of a more comprehensive approach to psoriasis treatment, as obese patients with moderate-to-severe psoriasis had a better response to low-dose cyclosporine A if a calorie-reduced diet was included in their treatment regimen (134).

### Prevention

The state of chronic systemic inflammation in psoriasis may at least contribute to atherosclerosis *via* insulin resistance and endothelial dysfunction and therefore be partially responsible for the increased cardiovascular risk of patients with severe psoriasis. This raises the question whether continuous systemic anti-inflammatory treatments may help to reduce this excess risk. Recently, data from the CANTOS trial suggest that this is principally feasible. That trial evaluated the efficacy of the interleukin-1β-blocking antibody canakinumab in more than 10,000 patients with previous myocardial infarction and a high-sensitivity C-reactive protein level of 2 mg or more per liter on nonfatal myocardial infarction, nonfatal stroke, or cardiovascular death. At a median follow-up of 3.7 years, the incidence rate was 4.50 events per 100 patient-years in the placebo-group, and between 3.86 and 4.11 events per 100 patient-years in the different canakinumab dosing groups. Thus, the trial documented a small, but statistically significant reduction of recurrent cardiovascular events in a high-risk population (135).

An early hint that continuous systemic anti-inflammatory treatment might reduce the cardiovascular risk of psoriasis came from a retrospective study by Prodanovich et al. who analyzed the files of more than 7,000 American veterans who had been treated over extended periods of time with methotrexate for their psoriasis. They found a significantly reduced incidence of cardiovascular diseases in those patients (136). Since then, several observational studies analyzing the effects of methotrexate or TNF-α inhibitors came to similar conclusions (137, 138), while others failed to document such protective effects (139, 140). Complementary to these studies, small controlled trials were performed, evaluating changes of biomarkers for cardiovascular risk under systemic anti-psoriatic treatment. Indeed, several groups reported amelioration of such markers under successful therapy. These include cytokines, adipokines, endothelial dysfunction, and carotid intima-media thickness (141–145).

Based on some of these observations, more ambitious projects were launched, sponsored by pharmaceutical companies. A pilot study on 30 psoriasis patients looked at vascular inflammation in the ascending aorta and carotid arteries by means of PET-CTs. In this study, decreases in vascular inflammation were observed in patients treated with adalimumab compared with placebo when data for the ascending aorta and carotid arteries were analyzed separately at 15 weeks (146). A larger study including 107 patients showed no difference over 16 weeks in the adalimumab-treated group compared to placebo and a modest increase in vascular inflammation in the carotid arteries after 52 weeks of treatment with adalimumab (147). A commentary on that publication suggested that the study might have been too small or of insufficient duration to show an effect, and that it was the carotid arteries and ascending aorta that were studied, not the coronary arteries, which might explain the negative result (20). Another study using the IL-17A-blocking antibody secukinumab was performed in a multi-center setting in Germany. A recently presented abstract on the occasion of the congress of the European Academy for Dermatology and Venereology in Geneva in 2017 documented a trend toward improvement in vascular elasticity, but no date from that trial have so far been published in a peer-reviewed manner.

Taken together, there is some evidence in favor of the idea to reduce the excess cardiovascular risk of psoriasis patients through systemic anti-inflammatory therapy. TNF-α and IL-17A are interesting targets in this regard, as the former is an important insulin antagonist, and the latter seems to exhibit primarily pro-atherogenic effects, although some anti-atherogenic effects have also been reported (148). The CANTOS study can serve as proof of concept for the principal capacity of anti-inflammatory therapy to reduce the cardiovascular risk, but it also points out that even in a high-risk population the effect size might be relatively small. This may explain why much smaller and shorter studies can easily fail to show efficacy in this regard, even more so, if relatively health patients are enrolled, as has been the case in the German trial using secukinumab. Although this approach remains intellectually appealing, it may be more effective and efficient in a real-world scenario to address other major cardiovascular risk factors associated with psoriasis in order to reduce the patients’ excess cardiovascular risk, e.g., through appropriate treatment of the metabolic syndrome or components thereof, or through lifestyle interventions (smoking!).

## Conclusion

Psoriasis is currently regarded a chronic-recurrent, systemic inflammatory disease, driven by an intense cross talk between cells of the innate and adaptive immune system. It is associated with substantial cardiovascular comorbidity, which can only partially be explained through shared a genetic control. Evidence in favor of psoriasis as an independent cardiovascular risk factor comes from epidemiologic studies showing a “dose effect” of psoriasis on the patients’ cardiovascular risk as well as from case–control studies looking at biomarkers for cardiovascular risk and specific manifestations of atherosclerosis. Inflammation-induced insulin resistance and endothelial dysfunction provide a pathogenetic link between psoriasis and atherosclerosis. Whether systemic anti-inflammatory therapy can reduce the patients’ excess cardiovascular risk remains one of the hot research topics in the field. Independent of this discussion, a comprehensive approach to the management of psoriasis at least in patients with severe disease is mandatory based on current knowledge. This must include regular screening and monitoring of traditional cardiovascular risk factors as well as their guideline-oriented treatment.

## Author Contributions

W-HB is the sole author of this manuscript. He defined the content, performed the literature search, wrote the text, and created figures and the table.

## Conflict of Interest Statement

The author received honoraria as a speaker or advisor from the following companies: Abbvie, Almirall, BMS, Celgene, Janssen, Leo, Lilly, Novartis, Sun Pharmaceuticals, and UCB. The author has received a research grant to study the role of Janus kinases in the pathogenesis of psoriasis from Pfizer. The author is supported by a grant from The Swiss National Foundation (310030_175470/1).
